# Doença Arterial Coronariana e Doença Renal Crônica: Uma História de Dois Órgãos

**DOI:** 10.36660/abc.20260028

**Published:** 2026-03-03

**Authors:** Igor Kranjec

**Affiliations:** 1 eFESC University Medical Centre Ljubljana Ljubljana Eslovênia eFESC University Medical Centre Ljubljana – Cardiology, Ljubljana – Eslovênia

**Keywords:** Doença da Artéria Coronariana, Insuficiência Renal Crônica, Prognóstico

"Era o melhor dos tempos, era o pior dos tempos…" Assim começa Um Conto de Duas Cidades, de Charles Dickens, um romance profundamente enraizado em Londres, enquanto lança um olhar atento através do Canal da Mancha para a turbulência da Paris revolucionária. Em contraste com a narrativa de divisão de Dickens, os dois órgãos vitais no centro deste editorial não existem em conflito, mas em sinergia. O coração e os rins trabalham em conjunto para manter a homeostase; quando um falha, o outro inevitavelmente o segue. As anormalidades cardiovasculares na doença renal crônica (DRC) são impulsionadas por uma interação complexa de fatores de risco tradicionais e estressores não tradicionais, específicos da DRC. À medida que a taxa de filtração glomerular (TFG) diminui, as manifestações clínicas da doença arterial coronariana (DAC) tornam-se cada vez mais prevalentes. Pacientes com DAC e DRC concomitantes representam um subgrupo distinto e desafiador.^1^ Eles frequentemente apresentam sintomas atípicos e são mais propensos a sofrer infarto do miocárdio sem supradesnivelamento do segmento ST do que angina estável de esforço. Além disso, a morte súbita cardíaca é um desfecho tragicamente comum na doença renal em estágio terminal.

A clareza diagnóstica é frequentemente difícil de alcançar nessa população. As troponinas cardíacas estão frequentemente elevadas cronicamente, mesmo na ausência de síndrome coronariana aguda, embora os mecanismos exatos ainda sejam debatidos. Além disso, o rendimento diagnóstico dos testes de esforço e da cintilografia de perfusão miocárdica é reduzido, levando a uma maior incidência de resultados falso-negativos e falso-positivos. Embora a calcificação coronariana seja onipresente na DRC e tenha peso prognóstico semelhante ao da população em geral, sua progressão é significativamente acelerada à medida que a função renal se deteriora. Estudos histológicos revelam ainda uma maior carga inflamatória nas placas coronarianas em comparação com controles sem doença renal. Apesar dessas complexidades, os pacientes com DRC permanecem sistematicamente sub-representados em ensaios clínicos, deixando uma lacuna de diretrizes baseadas em evidências sobre o uso de estatinas, revascularização eletiva e a duração ideal da dupla terapia antiplaquetária. A avaliação da DAC tradicionalmente se baseia na avaliação clínica integrada a ferramentas diagnósticas como eletrocardiografia, teste de esforço e ecocardiografia. Apesar de suas limitações conhecidas, a estenose luminal coronária permanece o parâmetro convencional para a gravidade da DAC na prática diária, orientando as decisões de tratamento após angiografia coronária invasiva ou angiotomografia computadorizada (angioTC).^2^ No entanto, evidências emergentes sugerem que a carga de placa, e não apenas o grau de estenose, é o principal preditor de risco cardiovascular, independentemente de a DAC ser obstrutiva ou não obstrutiva.^3^ A angioTC permite uma caracterização abrangente dessa carga e a identificação de características de placa de alto risco. Além disso, a incorporação de métodos de reserva de fluxo fracionada para considerar a DAC difusa pode refinar a avaliação anatômica da isquemia específica do vaso. A TFG permanece a métrica definitiva da função renal.^4^ Embora o "padrão ouro" envolva a medição da depuração de marcadores exógenos como a inulina, essa abordagem é muito invasiva, demorada e dispendiosa para uso rotineiro. Consequentemente, a prática clínica depende de estimativas da taxa de filtração glomerular TFG derivadas da creatinina ou da cistatina C. A precisão dessas estimativas é frequentemente comprometida por determinantes da creatinina que não estão relacionados à TFG, como dieta, massa muscular, secreção tubular e eliminação extrarrenal. Mesmo em populações bem validadas, 10% dos pacientes podem apresentar erros de estimativa superiores a 30%. A equação de Cockcroft-Gault (CG) compartilha as falhas inerentes à depuração de creatinina medida. A equação de Modificação da Dieta na Doença Renal (MDRD) é amplamente utilizada, particularmente em pacientes com insuficiência renal avançada, porém perde precisão naqueles com função renal preservada.^5^ Em contraste, a equação CKD-EPI (Colaboração em Epidemiologia da Doença Renal Crônica) — que utiliza as mesmas variáveis que a MDRD — oferece precisão superior em níveis mais altos de TFG, reduzindo o viés sistemático. Assim,A equação CKD-EPI é agora o padrão internacionalmente recomendado para a prática laboratorial de rotina.

A função renal é um potente preditor de desfechos cardiovasculares. A análise da coorte NHANES (n=31.020) demonstrou uma relação inversa não linear entre a redução da TFG e o risco de DAC, caracterizada por claros efeitos de limiar e saturação.^6^ A equação MDRD, em particular, emergiu como uma ferramenta prognóstica robusta, correlacionando-se com o número de vasos estenóticos,^7,8^ a complexidade da doença (escore Syntax),^9^ a carga de placa angiográfica^10^ e as apresentações iniciais de infarto do miocárdio.^11^ Nesta edição do periódico, Verdoia et al. apresentam um estudo de coorte em larga escala comparando as três principais fórmulas de TFG (CG, MDRD e CKD-EPI) em sua capacidade de predizer a prevalência e a extensão da DAC definida angiograficamente.^12^ Curiosamente, eles descobriram que apenas a fórmula MDRD permaneceu significativamente associada à gravidade da DAC. Por meio da análise da curva ROC, identificaram uma TFG de 12,4 ml/min/1,73 m² como o limiar ideal para prever DAC grave. DAC e DRC estão inextricavelmente ligadas em uma relação bidirecional que permanece apenas parcialmente compreendida. A busca pelo marcador "ideal" da função renal continua; até lá, a estimativa da TFG mais apropriada deve ser adaptada ao contexto clínico. Da mesma forma, a avaliação da DAC deve ser personalizada, empregando métodos não invasivos que capturem tanto a carga anatômica quanto a significância funcional. O manejo deve ser holístico, preenchendo a lacuna entre a prevenção cardiovascular e a nefroproteção rigorosa.
