# Supplementary material for: Doença Arterial Coronariana e Doença Renal Crônica: Uma História de Dois Órgãos
Source: Arq Bras Cardiol. 2026 Mar 3;123(1):e20260028. [Article in Portuguese] doi: 10.36660/abc.20260028 (PMC13127812; doi:10.36660/abc.20260028)
Supplement: Translation

# Translation: Coronary Artery Disease and Chronic Kidney Disease: A Tale of Two Organs

**DOI:** 10.36660/abc.20260028i

**Authors:** Igor Kranjec

**Affiliations:** 1 Ljubljana Slovenia eFESC University Medical Centre Ljubljana – Cardiology, Ljubljana – Slovenia

**Keywords:** Coronary Artery Disease, Chronic Renal Insufficiency, Prognosis

"It was the best of times, it was the worst of times…" So begins Charles Dickens's *A Tale of Two Cities*, a novel deeply rooted in London while casting a watchful eye across the Channel toward the upheaval of revolutionary Paris. In contrast to Dickens's narrative of division, the two vital organs at the heart of this Editorial exist not in conflict, but in synergy. The heart and the kidneys work in tandem to maintain homeostasis; when one falters, the other inevitably follows. Cardiovascular abnormalities in chronic kidney disease (CKD) are driven by a complex interplay of traditional risk factors and non-traditional, CKD-specific stressors. As the glomerular filtration rate (GFR) declines, the clinical manifestations of coronary artery disease (CAD) become increasingly prevalent. Patients with concomitant CAD and CKD represent a distinct and challenging subgroup.^1^ They frequently present with atypical symptoms and are more likely to experience non-ST-segment myocardial infarction than stable exertional angina. Furthermore, sudden cardiac death is a tragically common endpoint in end-stage kidney disease.

Diagnostic clarity is often elusive in this population. Cardiac troponins are frequently chronically elevated even in the absence of acute coronary syndrome, though the exact mechanisms remain debated. Moreover, the diagnostic yield of exercise testing and pharmacologic perfusion imaging is diminished, leading to a higher incidence of both false-negative and false-positive results. While coronary calcification is ubiquitous in CKD and carries prognostic weight similar to that in the general population, its progression is significantly accelerated as renal function deteriorates. Histological studies further reveal a higher inflammatory burden within coronary plaques compared to non-renal controls. Despite these complexities, CKD patients remain systematically under-represented in clinical trials, leaving a vacuum of evidence-based guidelines regarding statin use, elective revascularization, and the optimal duration of dual antiplatelet therapy. The evaluation of CAD traditionally relies on clinical assessment integrated with diagnostic tools such as electrocardiography, stress testing, and echocardiography. Despite its known limitations, coronary luminal stenosis remains the conventional benchmark for CAD severity in daily practice, guiding management decisions following invasive coronary angiography or computed tomography angiography (CTA).^2^ However, emerging evidence suggests that plaque burden, rather than the degree of stenosis alone, is the primary predictor of cardiovascular risk, regardless of whether the CAD is obstructive or non-obstructive.^3^ CTA allows for an extensive characterization of this burden and the identification of high-risk plaque features. Furthermore, incorporating fractional flow reserve methods to account for diffuse CAD may refine the anatomical assessment of vessel-specific ischemia. GFR remains the definitive metric of kidney function.^4^ While the »gold standard« involves measuring the clearance of exogenous markers like inulin, this approach is too invasive, time-consuming, and costly for routine use. Consequently, clinical practice relies on GFR estimates (eGFR) derived from creatinine or cystatin C. The accuracy of these estimates is often compromised by non-GFR determinants of creatinine, such as diet, muscle mass, tubular secretion, and extrarenal elimination. Even in well-validated populations, 10% of patients may have estimation errors exceeding 30%. The Cockcroft-Gault (C-G) equation shares the inherent flaws of measured creatinine clearance. The Modification of Diet in Renal Disease (MDRD) equation is widely utilized, particularly in patients with advanced renal impairment, yet it loses precision in those with preserved kidney function.^5^ In contrast, the CKD-EPI (Chronic Kidney Disease Epidemiology Collaboration) equation—which utilizes the same variables as MDRD—offers superior accuracy at higher GFR levels by reducing systematic bias. Accordingly, the CKD-EPI equation is now the internationally recommended standard for routine laboratory practice.

Renal function is a potent predictor of cardiovascular outcomes. Analysis of the NHANES cohort (n=31,020) demonstrated a non-linear inverse relationship between GFR decline and CAD risk, characterized by clear threshold and saturation effects.^6^ The MDRD equation, in particular, has emerged as a robust prognostic tool, correlating with the number of stenotic vessels,^7,8^ the complexity of disease (Syntax score),^9^ angiographic plaque burden,^10^ and the initial myocardial infarction presentations.^11^ In this issue of the Journal, Verdoia et al. present a large-scale cohort study comparing the three primary GFR formulas (C-G, MDRD, and CKD-EPI) in their ability to predict the prevalence and extent of angiographically defined CAD.^12^ Interestingly, they found that only the MDRD formula remained significantly associated with CAD severity. Through ROC curve analysis, they identified a GFR of 12.4 ml/min/1.73 m² as the optimal threshold for predicting severe CAD. CAD and CKD are inextricably linked in a bidirectional relationship that remains only partially understood. The search for the »ideal« marker of kidney function continues; until then, the most appropriate GFR estimation must be tailored to the clinical context. Similarly, CAD assessment should be personalized, employing non-invasive methods that capture both anatomical burden and functional significance. Management must be holistic, bridging the gap between cardiovascular prevention and rigorous nephroprotection.
